# An ancient competition for the conserved branchpoint sequence influences physiological and evolutionary outcomes in splicing

**DOI:** 10.1101/2024.10.09.617384

**Published:** 2024-10-09

**Authors:** Karen Larissa Pereira de Castro, Jose M. Abril, Kuo-Chieh Liao, Haiping Hao, John Paul Donohue, William K. Russell, W. Samuel Fagg

**Affiliations:** 1 Transplant Division, Department of Surgery, University of Texas Medical Branch, Galveston, TX, USA.; 2 RNA Genomics and Structure, Genome Institute of Singapore, Agency for Science, Technology, and Research (A*STAR) Singapore.; 3 Department of Biochemistry and Molecular Biology, University of Texas Medical Branch, Galveston, Texas, USA.; 4 Sinsheimer Labs, RNA Center for Molecular Biology, Department of Molecular, Cell and Developmental Biology, University of California, Santa Cruz, Santa Cruz, CA, USA.

**Keywords:** alternative splicing, branchpoint, Quaking, RNA binding protein, splicing, Splicing Factor 1 (SF1)

## Abstract

Recognition of the intron branchpoint during spliceosome assembly is a multistep process that defines both mRNA structure and amount. A branchpoint sequence motif UACUAAC is variably conserved in eukaryotic genomes, but in some organisms more than one protein can recognize it. Here we show that SF1 and Quaking (QKI) compete for a subset of intron branchpoints with the sequence ACUAA. SF1 activates exon inclusion through this sequence, but QKI represses the inclusion of alternatively spliced exons with this intron branchpoint sequence. Using mutant reporters derived from a natural intron with two branchpoint-like sequences, we find that when either branchpoint sequence is mutated, the other is used as a branchpoint, but when both are present, neither is used due to high affinity binding and strong splicing repression by QKI. QKI occupancy at the dual branchpoint site directly prevents SF1 binding and subsequent recruitment of spliceosome-associated factors. Finally, the ectopic expression of QKI in budding yeast (which lacks *QKI*) is lethal, due at least in part to widespread splicing repression. In conclusion, QKI can function as a splicing repressor by directly competing with SF1/BBP for a subset of branchpoint sequences that closely mirror its high affinity binding site. This suggests that *QKI* and degenerate branchpoint sequences may have co-evolved as a means through which specific gene expression patterns could be maintained in QKI-expressing or non-expressing cells in metazoans, plants, and animals.

## Introduction

Up to 95% of human protein-coding genes can be alternatively spliced (1, 2). In contrast, only a minority of *S. cerevisiae* genes contain introns, and under normal growth conditions they are efficiently spliced (3, 4). Many influences converge to promote extensive alternative splicing that is observed in higher eukaryotes including genome complexity, *cis*-acting regulatory elements, and *trans*-acting factors, (5–7). An early post-transcriptional step that is required for splicing is branchpoint (bp) recognition, which, along with 5’ splice site (ss), polypyrimidine (pY) tract, and 3’ss recognition, ultimately define the spliceosome E-complex (8, 9). Sequence variability in these elements can influence how efficiently E-complex and subsequent splicing complexes are able to mature (10–15). An interesting bp sequence-specific observation is that different organisms maintain conservation of it; for example in *Saccharomyces cerevisiae* (*S. cerevisiae*) it is nearly invariant (UACUAAC) while in mammals it is more degenerate (YUNAY) (16–19). The underlying functional issues that explain this variability in bp sequence (bps) conservation are unknown, however degeneracy is observed to a higher degree in organisms with more alternative splicing. This suggests that bps variability might provide a means through which alternative splicing can be controlled, but how this could be mediated is unclear.

RNA binding proteins (RBPs) can influence the recognition of the bps by SF1 (mammals)(20) or MSL5/BBP (*S. cerevisiae*)(14, 21). When these and other E-complex-associated proteins and snRNAs associate with the substate, subsequently SF1/BBP evicted and then replaced by the SF3B complex which recruits the 17S U2 snRNP, signaling maturation into the spliceosome A complex (22–24). Disruption of these events by either mutations or non-physiological concentrations of RBPs can lead to splicing defects and disease (25). For example, the balance between splicing-activating serine-arginine rich (SR) proteins versus splicing-repressing heterogenous nuclear ribonuclear proteins (hnRNPs) regulates a large set of pre-mRNA substrates, and provides an example of how competition between RBPs regulates alternative splicing (26, 27). It is not clear, however, if RBP competition can influence splicing in a cell type-specific manner, or if it might impact the evolutionary landscape.

**Figure 1:**
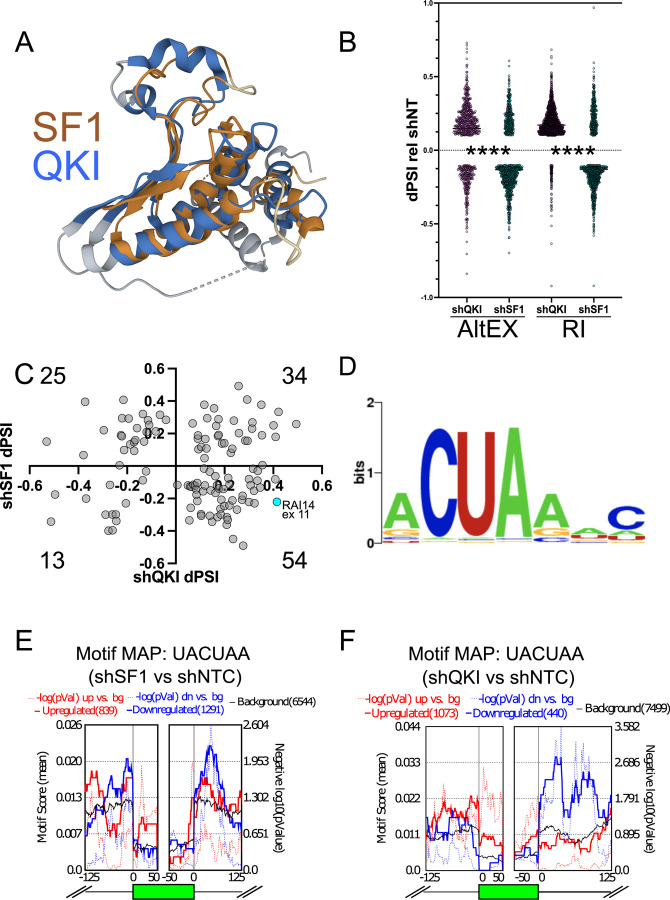
Similarity of SF1 and QKI and RNA-seq analysis of SF1 or QKI loss-of-function in HepG2 cells. A. Overlay of SF1 and QKI KH and QUA2 protein domains. B. Vast-tools analysis of the ENCODE RNA-seq data from SF1 (shSF1) or QKI shRNA (shQKI) knockdown compared to control shRNA (shNT) in HepG2 cells; y-axis shows dPSI fpr shQKI or shSF1 relative to shNT for significantly altered alternatively spliced exons (AltEX; left) or retained introns (RI; right; dPSI ≥ |10| and MVdPSI > 0), and ****P < 0.0001 by Mann-Whitney U when comparing distribution of changes in shQKI relative to shNT to shSF1 relative to shNT). C. Scatter plot showing the distribution of AltEX events that changed under depletion of both SF1 (dPSI values relative to shNT on y-axis) and QKI (dPSI values relative to shNT on x-axis); the number in each quadrant indicates how many AltEX events were observed; RAI14 exon 11 is shown in cyan. D. Simple Enrichment Analysis (SEA) of the intron region spanning 60 nt to 20 nt upstream of the 3’ss in QKI and SF1 regulated AltEX events shown in C (*P* < 0.05). E. rMAPS motif map for UACUAA generated for AltEX events changing during shSF1 compared to shNT by rMATS; motif scores (solid line) or −log10 P-value (dotted line) is shown in red for exons whose inclusion increases and in blue for exons whose inclusion decreases. F. rMAPS motif map as described in E. but for shQKI treatment compared to shNT.

The metazoan RBP Signal Transduction and Activator of RNA metabolism (STAR) family members possess a KH-type and QUA2 RNA binding domain and regulate disparate forms of RNA processing including splicing (28). Interestingly, SF1 is a unique, and the most divergent, member of the STAR family as it lacks the QUA1 dimerization domain that all other members possess (20, 29, 30). The QUA1-containing members like Quaking (QKI), KHDRBS1–3, GLD1, and ASD2 exist as dimers and thus bind to a bipartite sequence motif in their target RNAs (31, 32). QKI (33, 34) and SF1 (20) are structurally similar (Fig 1A), and although SF1 lacks the QUA1 dimerization domain, they bind to a similar sequence motif. Interestingly, the SF1 binding motif (35) is more degenerate than the QKI consensus motif (31, 36–39), which is consistent with SF1’s role in recognition of the more degenerate mammalian bps.

Analysis of tissue-specific splicing patterns nearly 20 years ago revealed that the UACUAAY motif is associated with muscle-specific exon skipping when located in the intron upstream of alternatively spliced exons or exon inclusion when located in the downstream intron, but the opposite pattern was observed in brain (40). It was unclear how the “conserved bp sequence” UACUAAY might promote exon skipping, or inclusion from the downstream intron, but suggested that this element may have an additional and specialized role in tissue-specific alternative splicing. Clarity was provided to this model with the discovery that QKI directly regulates pre-mRNA splicing in C2C12 myoblasts via binding an ACUAAY element and exhibiting the prototypical “splicing code” positionally-based regulatory pattern (38, 41, 42). This is also consistent with the findings that the nuclear isoform QKI5 is expressed at relatively high levels in muscle, but is lower in brain (38, 43–49). Thus, QKI5 enforces cell type-specific alternative splicing patterns by binding to a bp sequence that is also a high affinity QKI binding site to promote exon skipping.

Studies in lung/lung cells suggested that QKI could repress exon inclusion by competing with SF1 for a bps that might also function as a QKI binding site in the NUMB pre-mRNA (50). While this raised an intriguing possibility, it was unclear if these two RBPs were in direct competition for binding to a bona fide bps, and how competition for this specialized (but conserved consensus) bps might: affect global splicing patterns, modulate splicing in naturally-occurring intron substrates, alter spliceosome component recruitment, or influence evolutionary outcomes in splicing. Here we test these open questions and find that QKI5 (Qki5 in mouse) can repress a set of alternatively spliced exons whose bps mirror the high affinity QKI binding site. We use RAI14 exon 11 as a model and show that ACUAAC elements in intron 10 are true bps to which QKI or SF1 can bind to promote exon skipping or inclusion, respectively. Interestingly, QKI binding to RAI14 intron 10 bps leads to recruitment of paraspeckle-associated proteins, while SF1 binding causes subsequent enrichment of the SF3a/b and U2 17S complexes. These discoveries expand the scope of and provide clarity to the bp competition model. Moreover, the addition of QKI5 into *S. cerevisiae* is lethal, concomitant with widespread splicing repression. Together these findings suggest that the presence of *QKI* and degenerate bps may have co-evolved to expand the repertoire of cell type-specific alternative splicing in plants and multicellular eukaryotes.

## Results

### QKI and SF1 co-regulate a set of alternatively spliced exons through a distinct sequence motif

Based on analysis of their individual functions (14, 20, 35, 41, 51–55) and common structural features (Fig 1A), we hypothesized that QKI could repress the inclusion, while SF1 could activate the inclusion of a special subset of alternatively spliced exons that have ACUAA for their bps. To test this we analyzed RNA sequencing (RNA-seq) datasets during QKI or SF1 knockdown (HepG2 cells treated with control non-targeting shRNA or shRNAs targeting QKI (shQKI) or SF1 (shSF1)(56)) with VAST-tools to measure changes in alternative pre-mRNA splicing (57, 58). Consistent with potentially opposing functions in splicing, more exon inclusion and intron retention (IR) events are observed during QKI knockdown, but more exon skipping and fewer IR events are observed during SF1 knockdown; the distribution of these changes are significantly different when compared to one another (****P < 0.0001 by Mann-Whitney U; Fig 1B, Supplemental Fig S1, and Supplemental Table S1; significance cutoff: change in percent spliced in (dPSI) > |10| and minimum value dPSI at 95% confidence interval (MVdPSI95) > 0). To measure the distribution of exons regulated by both QKI and SF1 (co-regulated exons), we plotted dPSI values of cassette exons under each condition relative to control for those that change under both knockdown conditions (dPSI > |10| in either shQKI or shSF1 relative to control, and MVdPSI95 > 0 in both shQKI relative to control and SF1 relative to control). Interestingly, we observed that 54 out of the 126 (43%) of the co-regulated alternatively spliced exons fell into the category where inclusion increases upon QKI knockdown and decreases upon SF1 knockdown (Fig 1C). Thus the most highly represented set of these are QKI-repressed and SF1-activated. We next asked if these co-regulated alternatively spliced exons are associated with any significantly enriched sequences in the upstream intron region in which the bps is found. To do so, we obtained the sequence from 63 to 20 nucleotides upstream of the 3’ splice site (these should include putative bps) but exclude pY tracts and 3’ splice sites) of these co-regulated exons or 1000 control exons from transcripts that are expressed (base mean > 100) but whose splicing is unchanged upon either QKI or SF1 knockdown (dPSI < |1|, MVdPSI = 0) and used Simple Enrichment Analysis (SEA (59)) to test if any motifs are significantly enriched. This analysis revealed the enrichment of a single ACUAA-like motif for the co-regulated exons (*P* = 0.033), but failed to identify any significantly enriched motifs from the control sequences (Fig 1D). Interestingly, this motif could potentially serve as a QKI (31, 36, 37) or SF1 (35, 56) binding site, or bps (17, 18). Finally, splicing analysis of these RNA-seq datasets using the complementary method rMATS (60, 61) corroborated these findings by indicating more exon inclusion when QKI is knocked down, and more skipping when SF1 is reduced (Fig 1E and 1F; Supplemental Tables S2 and S3, respectively). Motif enrichment analysis using rMAPS2 shows significant enrichment of the UACUAA motif in introns upstream of exons that are skipped more when SF1 is reduced compared to control (Fig 1E), and enrichment of this motif in introns upstream of exons more included when QKI is reduced compared to control (Fig 1F). In summary, the most common of set of exons co-regulated by SF1 and QKI are SF1-activated and QKI-repressed, and these have enrichment of a bp-like sequence in their proximal upstream intron that also appears to be a QKI binding motif.

### RAI14 exon 11 is QKI-repressed and SF1-activated, with dual putative ACUAAC branchpoints

**Figure 2:**
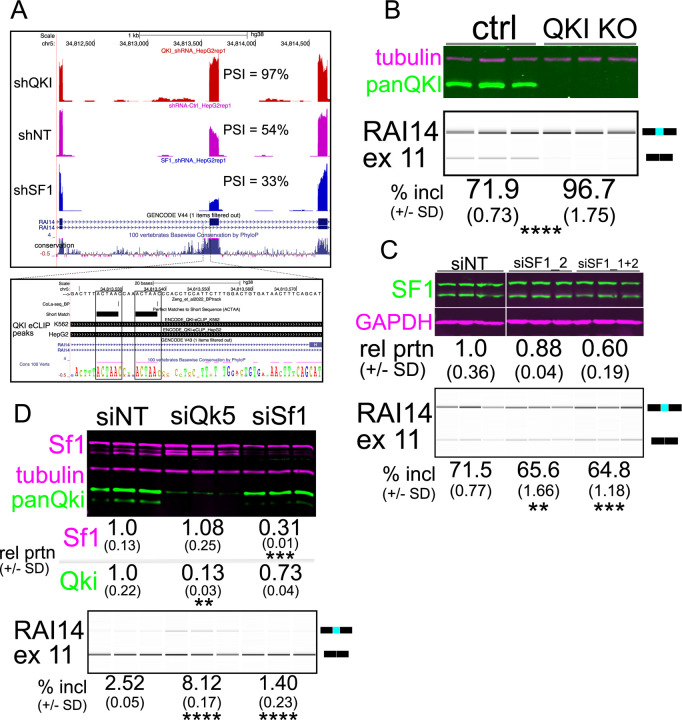
RAI14 exon 11 is repressed by QKI and activated by SF1. A. UCSC Genome Browser screenshot (top panel) with RNA-seq reads maping to RAI14 for shQKI (top), shNT (middle), or shSF1 (bottom); the inset shows boxed regions of (from top) two ACUAAC elements with Cola-seq branchpoints mapping to one nucleotide downstream of a branchpoint adenine, ACUAA elements by oligomatch, then QKI eCLIP peaks from K562 (top) or HepG2 (bottom) cells that overlap with these; conservation of 100 vertebrates is shown at the bottom. B. Western blot of protein extracted from HEK293 QKI KO cells (top) probed with anti-tubulin (magenta) or anti-panQKI (green) antibodies; RT-PCR of RAI14 exon 11 and BioAnalyzer gel-like image (bottom) of RNA extracted from the cells above showing mean percent included ± standard deviation below (n = 3 biological replicates; ****P < 0.001 by Student’s t-test compared to ctrl). C. Western blot (top) of proteins extracted from HEK293 cells transfected with siNT, siSF1_2 or siSF1_1+2, probed with anti-SF1 (green) or anti-Gapdh (magenta) antibodies; the protein abundance (fold change relative to the siNT control ± standard deviation) is shown below; RT-PCR of RAI14 exon 11 and BioAnalyzer gel-like image (bottom) of RNA extracted from the cells described above with mean percent included ± standard deviation below (n = 3 biological replicates; **P < 0.01 or ***P < 0.001 by Student’s t-test compared to siNT). D. Western blot (top) of proteins extracted from C2C12 myoblasts transfected with siNT, siQki or siSf1_1+2, probed with anti-SF1 (magenta; top), anti-tubulin (magenta; middle) or anti-panQki (green; bottom). The protein abundance (fold change relative to the siNT control ± standard deviation is shown below (n = 3 biological replicates; **P < 0.01 or ***P < 0.001 by Student’s t-test); RT-PCR of Rai14 exon 11 and BioAnalyzer gel-like image (bottom) of RNA extracted from the C2C12 cells described above with mean percent included ± standard deviation indicated below (****P < 0.0001 by Student’s t-test).

To further investigate how competition between QKI and SF1 for the bps is mediated, we sought a prototypical alternatively spliced exon subject to this form of regulation. The criteria by which we narrowed our search were 1) a QKI-repressed and SF1-activated cassette exon with 2) experimental evidence of a functional ACUAAY bp, and 3) experimental evidence of direct QKI binding. We previously found that Rai14 exon 10 is repressed by Qki5 and that reads from Qki iCLIP-seq map to the intron region just upstream of this exon in mouse myoblasts (38). Its inclusion is also repressed by QKI and activated by SF1 in HepG2 cells (Fig 1C (cyan dot) and Fig 2A; (56)). Inspection of the upstream intron region proximal to RAI14 exon 11 revealed intriguing features that fulfilled the above criteria: tandem ACUAAC elements 34 nt or 43 nt upstream of the 3’ss, experimental evidence indicating that either of these could be used as a bp (18), and QKI eCLIP peaks from both HepG2 and K562 cells (56, 62) that overlap with these putative ACUAAC bp sequences (Fig 2A). We next tested if the co-regulation of RAI14 exon 11 splicing by QKI and SF1 observed in HepG2 cells could be observed in additional cell types. We measured RAI14 exon 11 inclusion in HEK293 QKI KO cells and found a significant increase in RAI14 exon 11 inclusion in the KO cells compared to WT (*P* < 0.0001 by Student’s t-test; Fig 2B). To reduce SF1 levels, we used two independent siRNAs targeting it in WT HEK293 cells. The first failed to produce an appreciable knockdown, but the second or the two combined reduce the SF1 protein level, which causes more RAI14 exon 11 skipping (Fig 2C). Overexpression of myc:Qki5 but not a myc:Qki5 construct with reduced RNA binding activity (K120A;R124A (33)) promotes skipping of RAI14 exon 11 in WT HEK293 cells, and overexpressing SF1 causes more inclusion (Fig S2). Although the physiological level of Rai14 exon 11 inclusion is low in mouse myoblasts (perhaps due to the high level of Qki5 in these cells), knocking down Qki5 or Sf1 in C2C12 cells leads to a significant increase or decrease, respectively, in its inclusion compared to control (*P* < 0.0001 by Student’s t-test; Fig 2D). Therefore, RAI14 exon 11 splicing is repressed by QKI and activated by SF1 in various cell types, possibly by binding dual ACUAAC bps elements.

### Efficient RAI14 exon 11 skipping requires tandem ACUAAC elements, either of which can be used as a bp

**Figure 3:**
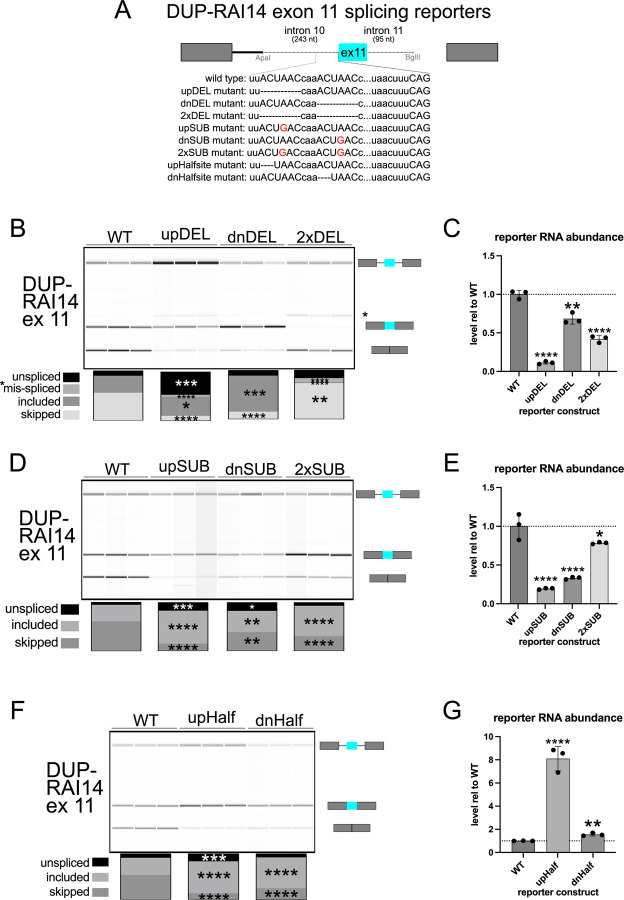
Analysis of DUP-RAI14 exon 11 (ex 11) splicing reporter. A. Schematic of the beta globin pDUP-RAI14 ex 11 splicing reporters indicating the region of intron 10, exon 11, and intron 11 included; the inset below show the different constructs (wild type or mutant) tested. B. RT-PCR and BioAnalyzer gel-like image from RNA extracted from C2C12 cells transfected with RAI14 ex 11 wild type or deletion mutant reporters; the box plots below indicate the percent observed level of each splice variant shown (from top to bottom: unspliced RNA, *an unidentified/spurious product, the exon-included form, or exon-skipped form) with *P < 0.05, **P < 0.01, ***P < 0.001, or ****P < 0.0001. C. RT-qPCR measuring total reporter RNA level, normalized to Eef1a1, from RNA described in B., and shown as fold-change relative to WT (**P < 0.01 or ****P < 0.001 by Student’s t-test). D. RT-PCR and BioAnalyzer gel-like image from RNA extracted from C2C12 cells transfected with RAI14 ex 11 wild type or substitution mutant reporters, analyzed as described in B (*P < 0.05, **P < 0.01, ***P < 0.001, ****P < 0.0001). E. RT-qPCR measuring total reporter RNA level, normalized to Eef1a1, from RNA described in D., and shown as fold-change relative to WT (*P < 0.05 or ****P < 0.001 by Student’s t-test). F. RT-PCR and BioAnalyzer gel-like image from RNA extracted from C2C12 cells transfected with RAI14 ex 11 wild type or half-site mutant reporters, analyzed as described in B (***P < 0.001, ****P < 0.0001). G. RT-qPCR measuring total reporter RNA level, normalized to Eef1a1, from RNA described in F., and shown as fold-change relative to WT (**P < 0.01 or ****P < 0.001 by Student’s t-test). Each experiment was conducted in biological triplicate.

Next we asked how the ACUAAC elements in RAI14 intron 10 influence exon 11 alternative splicing and transcript stability. Previous investigation did not stringently discriminate between whether QKI and SF1 competed directly for binding to the bp sequence, or if dimeric QKI bound to a bipartite motif that flanked the bps and thus occluded SF1 binding to the true bp (50). We hypothesized that: 1) efficient exon 11 skipping/splicing repression requires two intact ACUAAC elements (or at least a single ACUAAC element and QKI “half-site” (which would constitute the previously SELEX-defined high affinity Quaking response element (QRE)) (31)), 2) one ACUAAC element or the other must be present for any exon inclusion (either can be a bps), 3) loss of both ACUAAC elements would lead to no exon inclusion (one or the other is required to have a bps), 4) conversion of ACUAAC elements to another (non-Qki binding motif) bps would lead to more inclusion; in regard to RNA stability: 5) removal of either ACUAAC element could destabilize the transcript, but 6) restoring either to a UAAC “half-site” could rescue this defect and promote exon skipping. To test these we generated a splicing reporter by cloning 243 nucleotides of RAI14 intron 10, RAI14 exon 11, and 95 nucleotides of RAI14 intron 11 into the beta globing splicing reporter pDUP51 (DUP-RAI14 exon 11). We first deleted either the first, the second, or both ACUAAC elements (Fig 3A), transfected these reporter plasmids into C2C12 cells, and then measured exon inclusion by RT-PCR or total transcript stability by RT-qPCR. Deletion of the upstream ACUAAC element leads to a slight increase in RAI14 exon 11 inclusion while deletion of the downstream element causes ~40% increase in inclusion (Fig 3B), indicating that both sites are required for strong splicing repression. Interestingly, we observe more unspliced RNA and the appearance of a mis-spliced product from the upstream deletion mutant compared to WT (Fig 3B); the latter may be due to the use of a weak/cryptic bp and 3’ss (see * on Fig 3B). Essentially no inclusion of RAI14 exon 11 is observed in the absence of both ACUAAC elements, except for the mis-spliced product noted above (Fig 3B). Control experiments show that these amplicons are reverse transcriptase-dependent, indicating that the unspliced products detected are not due to plasmid DNA contamination, and corroborate the results described above (Suppl Fig S3). Moreover, loss of the upstream ACUAAC element causes markedly reduced total reporter RNA levels; we observe a similar trend in the other deletion mutants, but to a lesser magnitude (Fig 3C). Therefore, potent splicing repression of RAI14 exon 10 requires both ACUAAC elements, and one or the other is required for any exon inclusion and thus they are bona fide bps.

We next asked if substitution of either ACUAAC element, or both, to ACU*G*AC (which should be a poor substrate for QKI binding but a suitable bp sequence (Fig 3A)) would also lead to increased inclusion of RAI14 exon 11 or influence reporter RNA stability. We observe a large increase in exon inclusion and decrease in skipping for either of the single substitution mutants and for the double substitution mutant (~20–35%; Fig 3D). More unspliced RNA is observed in either of the single substitution mutants as well (Fig 3D). As above, the amplification of these products is RT-dependent (Suppl Fig S3). Transcript stability is significantly reduced in either single substitution mutant (****P < 0.0001 by Student’s t-test), and is slightly lower in the double substitution mutant (Fig 3E). Finally, converting either ACUAAC element to a UAAC “half-site” results in comparable increases in inclusion/decreases in skipping compared to WT as described in the deletion or substitution mutants, and more RT-dependent (Suppl Fig S3) unspliced reporter in the upstream “half-site” mutant (Fig 3F). Interestingly, these “half-site” mutants are more stable than the WT reporter, especially in the upstream “half-site” mutant (Fig 3G). In summary, removal of either ACUAAC element or conversion to ACUGAC leads to splicing activation of RAI14 exon 11, indicating a requirement for dual ACUAAC elements for potent splicing repression.

### Qki binding to Rai14 intron 10 requires both ACUAAC elements and prevents spliceosome recruitment

**Figure 4:**
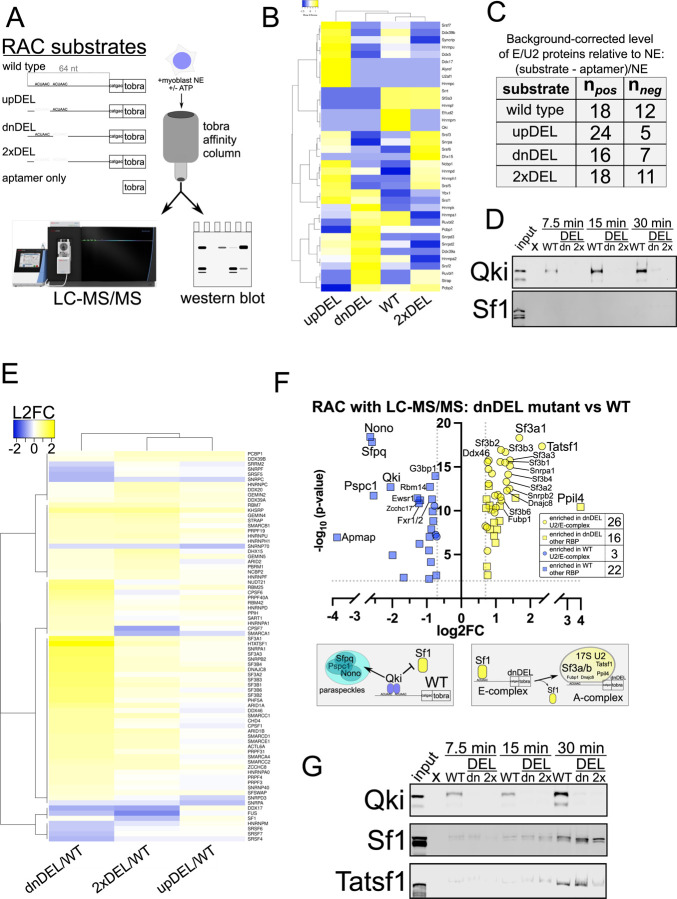
Analysis of the proteins associating with RAI14 intron 10 RNA. RNA affinity chromatography (RAC) with liquid chromatography and tandem mass spec or western blot analysis. A. Schematic representation of substrates used for RNA affinity chromatography (RAC) which included 64 nt of RAI14 intron 10, 6 nt of exon sequence, and the tobramycin aptamer (tobra): wild type, upDEL, dnDEL, 2xDEL and aptamer only; C2C12 nuclear extract (NE) was incubated with these, and RAC-associated eluates were analyzed by liquid chromatography with tandem mass spectrometry (LC-MS/MS) or western blot. B. Heatmap showing hierarchical clustering of early spliceosome and 17S U2 snRNP protein (E/U2) abundance detected in the RAC-LC-MS/MS datasets for each substate shown and in the presence of ATP; these represent background-corrected levels relative to NE (see Methods) and the scale bar shows row Z-score values. C. Numbers observed for relative levels of E/U2 proteins (background corrected relative to NE) observed associating with each RAC substate with either a positive (n_pos_) or negative (n_neg_) value. D. Western blot of NE (input) or WT, dnDEL (dn), or 2xDEL (2x) RAC time-course (+ATP as described in B and C) for 7.5 minutes (left), 15 minutes (middle), or 30 minutes (right) probed with anti-panQki (top) or anti-Sf1(bottom) antibodies. E. Heatmap showing hierarchical clustering of early spliceosome and 17S U2 snRNP protein (E/U2) abundance detected in the RAC-LC-MS/MS datasets for each substate shown and in the absence of ATP; these represent data-independent acquisition (DIA; see methods) values normalized to NE and each mutant is shown as log2 fold change relative to the WT substate and passed cutoff of log_2_ fold change > |0.2| and P < 0.01. F. Volcano plot comparing LC-MS/MS log_2_ protein abundance (log_2_ fold-change (log2FC); x-axis) of E/U2 (circles) and other RBPs (squares) observed associating with RAC substates dnDEL compared to WT (y-axis shows −log_10_ *P*-value) of enriched proteins (cutoff: L2FC > |0.7| and *P* < 0.01); inset shows the number observed for those enriched in dnDEL (yellow) or WT (blue); schematic below shows model of RAC substates recruitment to distinct protein-associated species. G. Western blot of NE (input) or WT, dnDEL (dn), or 2xDEL (2x) RAC time-course (-ATP as described in E and F) for 7.5 minutes (left), 15 minutes (middle), or 30 minutes (right) probed with anti-panQki (top), anti-Sf1 (middle), or anti-Tatsf1 (bottom).

We next hypothesized that Qki binding to Rai14 intron 10 requires both ACUAAC elements, that its binding would prevent spliceosome recruitment by blocking Sf1, and that removal of either ACUAAC element would favor Sf1 binding and recruitment of spliceosome components. To minimize bias, we initially performed RNA affinity chromatography (RAC) using 64 nt of intron sequence upstream of the 3’ss and including 6 nt of exonic sequence linked to a tobramycin aptamer (WT)(63), and the same sequence but with either the upstream ACUAAC (upDEL), downstream ACUAAC (dnDEL), or both ACUAACs (2xDEL), as well as a tobramycin aptamer (APT) only RNA (Fig 4A). Our rationale for this was to identify all proteins bound to these substates and correlate them with splicing outcomes: WT shows low levels of inclusion, upDEL slightly higher levels of inclusion but also more unspliced RNA, dnDEL high levels of inclusion, and 2xDEL lacks a bp and so is completely skipped. We added C2C12 nuclear extract (NE) to these under conditions that would favor splicing (with ATP) and then collected the associated proteins and identified them by liquid chromatography with tandem mass spectrometry (LC-MS/MS) (63). Quaking is readily identified associating with the WT RAC substrate and in the input NE, but is undetectable in each of the mutant RAC substrates and the APT only control; Sf1 is not detected associating with any RAC substrate but was present in NE (Fig 4B and 4C, and Supplemental Table S4). We used previously published LC-MS/MS datasets to generate a list of early spliceosome (E complex) and 17S U2 snRNP components (E/U2) (64–66) in order to focus on these proteins in our RAC-LC-MS/MS datasets to test our hypotheses. Subsequently we found that the proteomic profiles detected in association with the WT and 2xDEL substrates are the most similar (Fig 4B), suggesting that Qki protein binding or loss of bps lead to similar E/U2 protein binding patterns. Indeed, in either of the single deletion mutants we observe more enrichment of E/U2 components with the substrate RNA (24 positive and 5 negative values in upDEL and 16 positive values and 7 negative values in dnDEL) while the WT and 2xDEL substrates show a more balanced distribution (18 positive and 12 negative or 18 positive and 11 negative values, respectively; Fig 4C). Western blot analysis using the same RAC approach also shows robust Qki association with the WT substate, and undetectable or nearly undetectable Sf1 protein, validating our LC-MS/MS findings (Fig 4D).

Previous studies indicate that excluding ATP from RNA splicing/binding assays favors more stable association of early splicing complexes including Sf1 (12, 67, 68), so we performed RAC-LC-MS/MS with the same substrates in the absence of ATP (Supplemental Table S5). We also used a data independent acquisition (DIA) LC-MS/MS method in which we used NE to build a peptide search library in order to increase the specificity and sensitivity of detection (see Methods). We observe significant enrichment (log_2_ fold change > |0.2| and *P* < 0.01) of E/U2 components (including Sf1) binding to the RNA in the absence of the downstream ACUAAC (dnDEL) element compared to WT RNA (Fig 4E). This trend is also observed but to a lesser extent in the absence of the upstream ACUAAC (upDEL) and in the absence of both (2xDEL) mutants relative to WT; the proteome profiles associated to these two substrates are also more similar to one another than the dnDEL comparison to WT, consistent with lower splicing efficiency or no splicing, respectively (Fig 4E). Closer and more stringent (log_2_ fold change ≥ |0.7| and *P* < 0.01) inspection of the proteins binding preferentially to the dnDEL mutant RNA compared to the WT RNA reveal enrichment of 26 E/U2 components along with 16 other annotated (non-E/U2) RBPs; we observe depletion (or enrichment in WT) of only 3 E/U2 proteins but of 22 other annotated RBPs including Qki (Fig 4F). Interestingly, some of the most enriched protein components associating with the dnDEL RNA are Tatsf1, Sf3a1, Ppil4, Sf3a3, Sf3b4, Dnajc8, Sf3a2, Sf3b3, Sf3b1, and Fubp1 (Fig 4F) many of which are known to interact with the bps and Sf1 or are members of the Sf3 complex, which promotes spliceosome maturation by displacing Sf1 from the bp and then recruiting the U2 snRNP (24, 69–71). The most significantly enriched proteins associating with the WT RAC substate are Nono, Sfpq, Pspc1, and Qki; the former three are found in paraspeckles that are formed by liquid-liquid phase separation (72), and Qki has recently been identified as a paraspeckle component protein (73). Western blot analysis validates the above findings indicating that Qki associates with high affinity to the WT but not dnDEL or 2xDEL RNA, and, in the absence of ATP, Sf1 and Tatsf1 associate to a greater degree with the dnDEL mutant than either of the other RAC substates (Fig 4G). Therefore, both ACUAAC elements are required for Qki binding and repression of Rai14 exon 11 splicing; removing the downstream ACUAAC element causes Sf1 binding, and the subsequent recruitment of the spliceosome A complex machinery and de-repression of RAI14 exon 11 splicing.

### Ectopic expression of Qki5 in S. cerevisiae is lethal and causes pre-mRNA splicing defects

**Figure 5:**
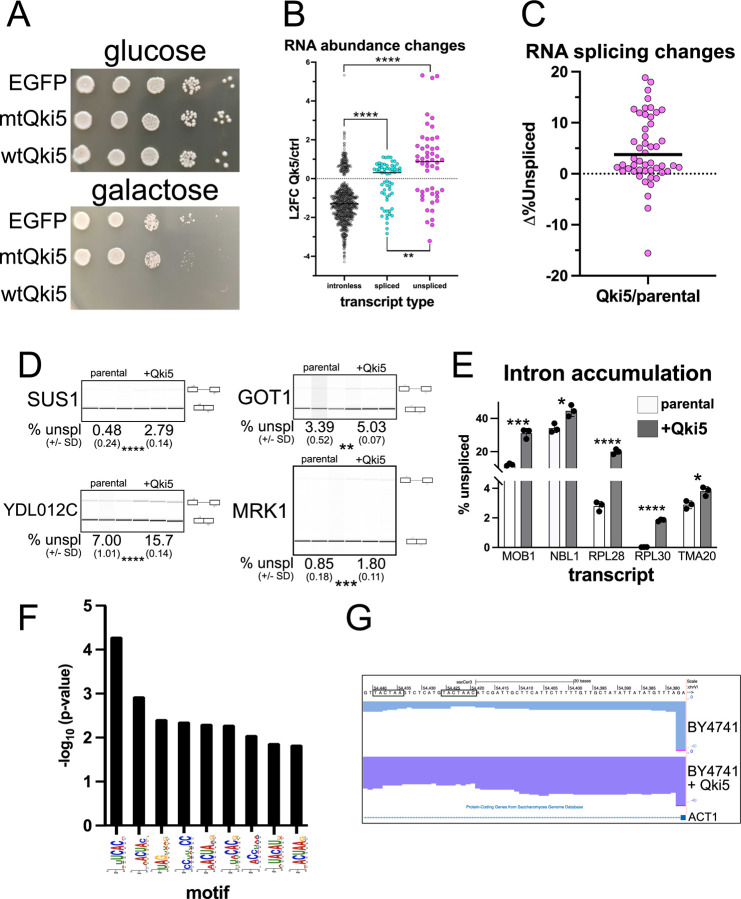
Qki5 expression is lethal in yeast and represses splicing. A. BY4741 *S. cerevisiae* strain with EGFP (top), mutant Qki5 (mtQki5; middle), or wild type Qki5 (wtQki5; bottom) GAL-inducible transgene cultures grown on either glucose- (top) or galactose-containing (bottom) YPD plates at decreasing densities (left to right) and incubated at 30°C for 72h. B. Changes in RNA abundance measured by Deseq2 analysis of RNA-seq data for intronless, spliced, or unspliced transcripts were measured from RNA extracted from BY4741 cells with Qki5 expression (induced by galactose-containing media for 4h), or in the BY4147 parental strain also cultured in galactose-containing media for 4h (control), and are shown as L2FC in Qki5-induced cells relative to control (n = 3 biological replicates; cutoff *P* < 0.1; abundance cutoff of TPM > 0.2); **P < 0.01 or ****P < 0.0001). C. Splicing changes measuring the change in percent unspliced (Δ%Unspliced; y-axis) for BY4741 as described in B.; cutoff *P* < 0.1 by Student’s t-test and base mean > 100. D. RT-PCR with primers that span exon-intron-exon junctions and BioAnalyzer gel-like image showing mean percent unspliced (+/− SD; n = 3) below for introns whose inclusion increased upon Qki5 ectopic expression and as measured by RNA-seq analysis in C. for the parental or Qki5-expressing cells (**P < 0.01, ***P < 0.001, ****P < 0.0001 by Student’s t-test). E. RT-qPCR analysis measuring mean percent unspliced transcript from RNA extracted from biological triplicate cultures of either the parental control or BY4741 expressing Qki5 for each transcript shown (+/− SD; *P < 0.05, ***P < 0.001, ****P < 0.001). F. Bar graph showing −log_10_ *P*-values (y-axis) of significantly enriched (SEA; *P* < 0.01) motifs observed in 3’ proximal ends of introns whose inclusion increases upon ectopic Qki5 expression in yeast (x-axis). G. UCSC Genome Browser screen shot showing RNA-seq reads mapping to the ACT1 transcript intron/exon junction near the 3’ss from RNA extracted from parental control and Qki5-induced cells; boxed sequences show two TACTAA elements.

Our results indicate that Qki and Sf1 can directly compete for a subset of ACUAA bp sequences, so we hypothesized that expressing the Qki5 isoform in *S. cerevisiae* (where the bp sequence is nearly invariant UACUAAC and lacks *QKI*) would be lethal and cause defective splicing. To test this, we inserted a galactose-inducible cDNA encoding either EGFP, mutant K120A;R124A Qki5, or wildtype Qki5 into BY4741 at the *URA3* locus, and each grew normally on glucose-containing media where the transgene is repressed. Galactose-induction is tolerated for the EGFP- and mutant Qki5-containing yeast, but is lethal for the wildtype Qki5-containing strain (Fig 5A). Growth curve analysis indicates that after about 4h the Qki5-expressing BY4741 cells cease proliferation (Supplemental Fig S5A). Interestingly, by 24h most of these cells have a “large-budded” morphology, which suggests a defect in cell division; EGFP- and mutant Qki5-expressing cells grow normally (Supplemental Fig S5A and S5B).

Next we tested how ectopic Qki5 expression impacts the yeast transcriptome. The parental BY4741 strain, or BY4741 with WT Qki5 were grown in galactose-containing media for 4h, and then we collected RNA and performed RNA sequencing and analysis. We used a *S. cerevisiae* genome annotation that specifies intronic regions and pre-mRNAs, spliced mRNAs, or intronless transcripts (74) to map RNA-seq reads to the transcriptome and then measured these different transcript types. We observe higher levels of unspliced pre-mRNA and spliced mRNA when comparing the changes in Qki5 induction to the control, while intronless transcript abundance decreases (cutoff *P* < 0.1; Fig 5B and Supplemental Table S6). Unspliced pre-mRNAs accumulate to a greater degree than spliced mRNAs, supporting the notion that ectopic Qki5 expression perturbs splicing in yeast (**P < 0.01 by Mann-Whitney U; Fig 5B). We measured changes in splicing by calculating the percentage of unspliced pre-mRNA for each intron-containing transcript (n = 304 (out of 325 total) that passed expression level cutoff of base mean > 100), and discovered that 50 change significantly (cutoff *P* < 0.1 by Student’s t-test; Supplemental Table S7) upon Qki5 expression. Of these, 41 increase in percent unspliced (~12% of all expressed intron-containing transcripts) while 9 decrease compared to control, which indicates a strong bias toward intron accumulation upon ectopic Qki5 expression (Fig 5C). We validated several of these using RT-PCR (Fig 5D (n = 4) and Supplemental Fig S5C (n = 15)) or RT-qPCR (Fig 5E (n = 5)) and found that each of these instances of repressed splicing upon Qki5 expression that is measured by RNA-seq is also observed using these complementary methods. Importantly, the HAC1 intron, which is not removed by the spliceosome (75), shows no increase in intron accumulation upon ectopic Qki5 expression by either RNA-seq or RT-PCR (Supplemental Fig S5C). Next we asked if any intron sequence motifs are enriched that correlate with increased intron retention upon Qki5 expression by obtaining 80 nt of intron sequence upstream of the 3’ss and compared these to 80 nt of intron sequence in 108 control introns that are expressed but unchanged compared to control using SEA ((59) see Methods). Nine motifs are significantly enriched (*P* < 0.01), 4 of which have varying degrees of resemblance to the QKI binding motif (ACUAA) or “half-site” (UAAY) (31, 36, 37). We note that several of these could potentially serve as tandem embedded motifs (for example, U*ACUA**A*CUAAC where the first is italicized and the second underlined). A search for these finds that 18% of the introns that accumulated upon Qki5 induction compared to control have either two independent or tandem embedded motifs. In contrast, examination of the introns that are unchanged upon Qki5 induction reveals that only 6% had two intron ACUAA motifs within 80 nt of the 3’ss. The occurrence of two ACUAA elements per intron 3’ss-proximal region is significantly overrepresented in the set of introns that accumulate upon Qki5 ectopic expression (*P* = 0.035 by Chi squared test), suggesting that these are more sensitive to Qki5 splicing repression. Intriguingly, the essential ACT1 pre-mRNA has dual ACUAA elements in its intron proximal to the 3’ss, similar to those observed in RAI14 intron 10 (Fig 2A), and its splicing is blocked by Qki5 induction (Fig 5F). Thus Qki5 expression in yeast is lethal and is due, at least in part, to splicing inhibition.

## Discussion

Our study reveals that QKI can repress the inclusion of alternatively spliced exons by directly competing with SF1/BBP for ACUAA bps. We find that 43% of the alternatively spliced exons that are co-regulated by both SF1 and QKI fall within the “QKI repressed and SF1 activated” category and that these are associated with an ACUAA sequence motif/bps (Figs 1 and 2). We show unambiguously that either of the two ACUAAC elements in RAI14 intron 10 can be used as a bps but that one or the other is required for any splicing, and so must be a bps (Fig 3). Interestingly, deleting either ACUAAC element reduces the level of exon skipping (Fig 3) and Qki5 binding (Fig 4), but increases the level of inclusion (Fig 3) and Sf1 binding (Fig 4). Therefore, Qki binds with high affinity to a bona fide dual bps in RAI14 intron 10 to prevent Sf1 binding and splicing activation. In budding yeast, where the bps in nearly invariant ACUAAC, ectopic expression of Qki5 blocks pre-mRNA splicing and is lethal (Fig 5). Together these findings demonstrate the validity of the bp competition model by showing these two RBPs directly compete for ACUAA bps substates.

### Consequences of bp competition for pre-mRNA substates and other RNAs

One of the most well-studied examples of competition between RBPs for pre-mRNA substrates is SR proteins that promote exon activation versus hnRNPs that repress exon inclusion (76, 77). This is the predominant manner through which most cell/tissue types carry out alternative pre-mRNA splicing. The model for bp competition between SF1 and QKI that we define here describes a novel but relative simple pathway through which a specific subset of alternatively spliced exons with ACUAA bps can be regulated. Our findings are consistent with the established role of SF1 in bp recognition and splicing activation, and suggest that QKI is more often a splicing repressor. A nuanced implication of this model is that loss-of-function of one of these proteins leads to a gain-of-function in the other, by relieving the competitive inhibition for a specific number of ACUAA bps that control the inclusion of certain alternatively spliced exons.

How does competition between SF1 and QKI influence transcript fate? In the case of RAI14 intron 10, the dual ACUAA bps constitute a high affinity bipartite binding site for dimeric Qki5 protein. In C2C12 myoblasts where Qki5 levels are high, Rai14 exon 11 is mostly skipped (Fig 2), and this correlates with Qki5 binding (Figs 2 and 4). This binding event appears to promotes paraspeckle association, as the paraspeckle proteins Nono, Sfpq, and Pspc1 (72) co-associate with Qki5-bound RAI14 intron 10 (Fig 4F). This is consistent with a recent report identifying QKI as a paraspeckle-associated protein (73). It is unclear how paraspeckle localization might influence splicing outcomes, but pre-mRNA localization within the nuclear speckle is associated with more efficient splicing (78). It is possible that QKI-directed paraspeckle localization is repressive to pre-mRNA splicing, or alternatively promotes the ligation of the exons flanking an alternatively spliced one, leading to exon skipping like we observe in RAI14. Further study is required to elucidate how competition between SF1 and QKI for pre-mRNA substates influences their subnuclear localization, splicing, and stability. A recent study suggests that this may be complicated by the fact that QKI itself attenuates paraspeckle biogenesis by promoting the expression of the short NEAT1 lncRNA isoform, which has lower paraspeckle-promoting activity than the longer isoform (79). It is unclear if SF1 might have a similar role, which is possible, given that QKI promotes expression of the short isoform of NEAT1 via ACUAA elements (to which SF1 might also bind). Therefore, this may constitute a complex feedback loop in which QKI (and/or SF1) can regulate splicing in a paraspeckle-dependent manner, but also regulates the degree to which paraspeckles accumulate.

Relatively little is known about how QKI or SF1 might regulate, or be regulated by, other lncRNAs. Each of these RBPs can interact with the lncRNA MIAT/Gomafu though, which has 16 ACUAA elements within about 1000 nt of RNA sequence (80, 81). While the functional impact of this is unclear, it is not difficult to imagine that they could compete for binding to this lncRNA (or vice-versa) within the same cell, and thus MIAT could function as a competing endogenous RNA (ceRNA) and attenuate splicing function. This could have a balancing effect on how the SF1/QKI axis influences splicing, if each were similarly affected. Alternatively, high MIAT levels might lead to preferential association with one protein over the other – perhaps QKI, given that its binding affinity is nearly two orders of magnitude higher than that of SF1 (14, 33) plus the transient nature of SF1 binding (12, 67) – this could effectively decrease the concentration of “free” QKI and skew bp competition more in favor of SF1 (and exon inclusion). This is termed trans-competition, and is widely applicable throughout Eukarya. For example in humans, the presence of many extra CUG repeats in the 3’UTR of the DMPK transcript is sufficient to sequester MBNL proteins (82, 83), causing a reduction-of-function in splicing which contributes to the development of myotonic dystrophy type I (84, 85). Therefore, trans-competition by influences from other RNAs such as MIAT/Gomafu, might also influence bps competition between SF1 and QKI.

### Bp competition defines a subset of cell type-specific alternative splicing

Competition for alternatively spliced exons between SR proteins and hnRNPs largely explains the splicing patterns observed in most cell types, but the most divergent patterns are observed in muscle or brain cells (86, 87). This is due in part to muscle- or brain-specific RBP expression patterns, and our study uncovers the novel finding that bp competition between SF1 and QKI explains in part how these are enforced. SF1 levels are relatively high in most tissue but can vary by up to 8-fold (58), while QKI levels are much more dynamic. QKI levels are elevated in muscle, but lower in total brain or predominated by the cytoplasmic isoforms Qki6 and Qki7 (45, 46, 48, 49). Therefore, in muscle the QKI:SF1 ratio is high, but in many brain cell types it is low, which will produce opposite patterns of isoform-specific transcriptomes and proteomes, within the context of the bp competition model. This is also relevant in developmentally-regulated systems where Qki5 levels increase during cardiac cell differentiation (88) but decrease as neural stem cells commit to mature cell types (39). Similarly, when embryonic stem cells exit pluripotency and commit to endoderm QKI levels go down, but QKI increases upon commitment to the mesodermal lineage and a QKI-associated splicing gain of function is observed (89). On the other hand, SF1 reduction, leading to increases in unspliced RNA and skipped exons, has been observed in a model of aging and correlates with age-related decline in fitness (90). In summary, bp competition between SF1 and QKI appears to be a novel molecular mechanism through which different cell type/tissue-, lineage-, developmental-specific, and homeostatic splicing programs can be achieved and regulated.

### Evolutionary implications of bp competition

Yeast and other single-celled eukaryotes lack *QKI*, but multicellular eukaryotes and plants have the gene or an orthologous one. Strikingly, we found that 12% of the introns expressed in yeast strain BY4741’s splicing was inhibited by ectopic Qki5 expression, and that these cells developed a large-budded phenotype due to a failure to divide (Supplemental Fig S5A and S5B). This phenotype has been observed due to lack of TUB1 pre-mRNA splicing (91), although we did not observe mis-splicing of it (or TUB3) in our study. The introns most sensitive to Qki5 have evidence of the “bipartite” QKI motif, which is consistent with high affinity binding of dimeric QKI (31) and similar to that observed in RAI14 intron 10. It is interesting that yeast lack *QKI* and do not exhibit “alternative splicing” per se, and also have a more invariant bps. This is UACUAAC in *S. cerevisiae* (14, 16, 19), but *Schizosaccharomyces pombe* have a slightly more degenerate bps (92), and its introns share some features with both budding yeast and mammals (93, 94). Nevertheless, the consensus bps in the latter still represents what would be a strong QKI binding motif (31, 36), suggesting it that QKI expression might also be lethal and thus it would be evolutionarily unfavorable to select for *QKI* or an ortholog/close relative. In contrast, one could envision a scenario in which single-celled organisms began evolving a genome that would later become that of a multicellular organism, potentiating the need to diversify cell type-specific functions in an energetically favorable manner. Co-evolving RBPs and their recognition motifs in order to generate different protein isoforms that can have cell type-specific function could have been a solution. This may be the case regarding the STAR family of RBPs, with exception to SF1/BBP, which is the most divergent and only member found in single-celled eukaryotes and lacking the QUA1 dimerization domain (30). In the case of QKI (or ASD2 in *C. elegans* (95) or HOW in *Drosophila* (96)), the binding motif ACUAA, along with the presence of a YAAY “half site” within about 20 nt, requires more specificity and generates additional points of contact on an RNA, which can explain in part its higher binding affinity than SF1. Perhaps it would not have been be evolutionarily advantageous for the majority of bps in multicellular organisms to be UACUAAC/high affinity QKI motifs (fewer than 20% of human introns have this bps (97)), and so more degenerate bps were selected for in metazoans. In contrast, selecting for some ACUAA bps could help diversify the function of cell type-specific protein isoforms. For example, many cytoskeletal/contraction-related protein isoforms are specific to muscle (98, 99) and many of these are directly regulated by QKI repressing inclusion of alternatively spliced exons through binding UACUAAY in introns upstream of them (38, 40, 41, 88). Similar observations in worms and flies suggest the intriguing possibility that the *QKI* gene and the more extensive bps degeneracy that is observed in metazoans and plants may have co-evolved. One manner in which this would have been executed but also constrained is through bps competition between QKI and SF1/BBP. Consequently, this may have allowed for expansion of the transcriptome and proteome, through the diversification of alternative splicing.

## Materials and Methods

### Cell culture

C2C12 mouse myoblasts and HEK293 cells were cultured in Dulbecco’s Modified Eagle Medium (DMEM) supplemented with high glucose (Invitrogen) and 10% heat-inactivated fetal bovine serum (Thermo Fisher). Cells were maintained at 37°C in a humidified atmosphere with 5% CO_2_. The HEK293 QKI KO cells were generated as previously described (100, 101).

### Plasmids and transfections

The pDUP51 splicing reporter plasmid (102) was used as a backbone to generate the RAI14 exon 11 plasmid. A 243 bp fragment upstream of exon 11, exon 11, and a 95 bp fragment downstream of the exon was PCR-amplified from H9 human embryonic stem cell genomic DNA; the forward primer contained an ApaI site and reverse primer contained a BglII site. These and the pDUP51 plasmid were digested with ApaI and BglII, then ligated into pDUP51 at the ApaI and BglII restriction sites. The resulting pDUP-RAI14 exon 11 plasmid was confirmed by Sanger sequencing. Mutant constructs were generated using the Q5^®^ Site-Directed Mutagenesis Kit and were also verified by Sanger sequencing. The myc:Qki5 plasmids used were described previously and were a kind gift from Sean Ryder (38, 89). Generation of the pcDNA3.1-tdTomato plasmid was also previously described (38). The pcDNA3.1-SF1 plasmid was generated by Gibson Assembly using cDNA from H9 human embryonic stem cells as template for PCR amplification and confirmed by Sanger sequencing.

Transfections were carried with Lipofectamine 2000 (Invitrogen) using a “in tube transfection” protocol, where 2.5X10^5^ cells were added to a tube containing Lipofectamine and the appropriate volume of reagent, following the manufacturer’s instructions, with 100 ng DNA mix in Gibco Opti-MEM (Thermo) or 30 pmol of siRNA, incubated for 20 minutes at RT, plated in 12 well plates containing DMEM 10% FBS and incubated overnight. Cells were harvested 24 hours after transfection.

For the RNA affinity chromatography, the j6f1 aptamer under the control of T7 RNA promoter was obtained by the digestion of the vector pcDNA5-aptamer plasmid, and was a gift from Chloe Nagasawa (University of Texas Medical Branch, Galveston, Texas, US) from the lab of Mariano Garcia-Blanco (University of Virginia). RAI14 sequence consisting of 60 bp fragment of RAI14 intron 10 upstream RAI14 exon 11 was obtained from a gBlock (IDT), digested with HindIII and Not1 restrictions enzymes and cloned into the digested PCNA5 aptamer plasmid.

For yeast transformation the plasmid pJW1666 (gift from Jonathan Weissman (Addgene plasmid # 112040; http://n2t.net/addgene:112040; RRID:Addgene_112040(103)) was used to clone the WT or mutant QKI sequences, or the GFP present on WT the plasmid was used as control. *RNA extraction, RT-PCR and RT-qPCR*

RNA was extracted from cells using Trizol (Thermo Fisher). For direct extraction from cell plates, Trizol was added to the wells and samples were vortexed. For extraction from cell lysates, cells were extracted with RSB100 buffer (100mM Tris-HCl pH 7.4, 0.5% NP-40, 0.5% Triton X-100, 0.1% SDS, 100 mM NaCl and 2.5 mM MgCl2). Samples were vortexed, chloroform was added then the samples were incubated at RT for 2 minutes. After centrifugation at 13,000 RPM for 15 minutes at 4°C, the aqueous phase was transferred to a new tube and samples were processed with the ReliaPrep RNA MiniPrep system (Promega), according to the manufacturer’s instructions. Reverse transcription was performed with iScript^™^ Reverse Transcription Supermix (BioRad) according to manufacturer instructions. RT-PCR was performed using Taq2x Master Mix (NEB) and cycle numbers were determined empirically to prevent over amplification. PCR products were analyzed by agarose gel and via Agilent 2100 BioAnalyzer.

For RT-qPCR cDNA was diluted 1:8 and used with Applied Biosystems^™^ SYBR^™^ Green Universal Master Mix on a Step one Plus Real time PCR machine (AppliedBiosystems). RT-qPCR cycling conditions were: 95°C for 10 minutes, [95°C 15 seconds, 60°C 60 seconds] (40 cycles). DUP-RAI14 exon 11 reporter RNA abundances were analyzed by calculating the ΔCt values normalized to EEF1A1. The ΔΔCt values were then calculated as 2^-ΔCt for each sample. The average ΔΔCt of the wild type reporter was used as a normalization factor to determine the relative RNA abundance in the mutant reporters.

### Western blotting

Proteins were extracted using RSB100 (100mM Tris-HCl pH 7.4, 0.5% NP-40, 0.5% Triton X-100, 0.1% SDS, 100 mM NaCl and 2.5 mM MgCl_2_) with EDTA-free protease inhibitor cocktail (SIGMAFAST, Sigma) and 1 mM PMSF. The buffer was added directly on the wells of the cell plates, scraped, collected, and kept on ice, while vortexing every five minutes for 30 minutes, followed by centrifugation at 14000 RPM for 15 minutes at 4°C. The supernatant was transferred to a new tube. Samples were stored at −80°C until use.

Protein concentration was measured using the Bradford assay (BioRad (104)). The same amount protein of each sample were loaded on 10% SDS-PAGE (typically 15–30 ug), and then transferred to 0.45 micron nitrocellulose membrane (Thermo). The membrane was blocked (with 5% non-fat dry milk dissolved in tris-buffered saline (TBS)) for one hour at room temperature with constant agitation. Primary antibody incubations were performed overnight at 4°C with constant agitation in TBS 0.01 tween (TBS-T) with 5% non-fat dry milk using the following antibodies: anti-panQKI, IgM-anti-GAPDH, α/β-Tubulin, anti-SF1 (A303-213a), and anti-HTATSF1, all diluted in TBS containing 5% milk and 0.01% Tween-20 (TBST). The following day, the membranes were washed three times with TBST with 5% milk at RT. Secondary infrared conjugated antibodies: IRDye 800CW Goat anti-Mouse IgG2b, IRDye 680RD Goat anti-Mouse IgM, IRDye 800CW Goat anti-Rabbit IgG and IRDye 680RD Goat anti-Rabbit IgG from Li-Cor were diluted according to manufacturer instructions in TBST with 5% non-fat dry milk for 1 hour with constant agitation. Membranes were washed 3 times every 5 minutes before visualization on Odyssey CLx imager (Li-Cor).

### Yeast transformation

Yeast transformation was performed as described by Ito *et al* with modifications (105) using the BY4741 strain. A 50 ml of culture of BY4741 was grown to a density of 5–10^6 overnight. Following centrifugation, the pellet was resuspended in 1ml of deionized water, centrifuged at 12000 RPM and resuspended in 0.5ml of 1X TE-LiAc (100mM tris-HCl, 10mM EDTA pH 7.5; LiAc pH 7.5). Subsequently cells were resuspended in 3x volume of 1X TE-LiAc and incubated at 30°C with agitation. 2 ug of purified PCR of WT QKI, mutant QKI and GFP plasmids and 7 ul of salmon sperm carrier DNA were added to 200 ul of the yeast cells and the mixture was incubated for 45 min at 30°C with agitation. The following steps included a 3 h incubation at 30°C with agitation, a 20 minute heat shock at 42°C, centrifugation at 12000 RPM and resuspension in YEPD followed by a 45 min incubation. After a 12000 RPM centrifugation, cells were resuspended in water and spread in URA-selection plates, then incubated at 30° C until transformants appear.

### Yeast genomic DNA extraction

Genomic DNA extraction was performed using the method developed by Klassen and collaborators with a few modifications (106). Cells were patched on URA-selection plates, and 50 ul of cell volume was scraped off the plate and resuspended in zymolase 20T 8 mg/ml. After vortexing the samples, they were incubated at 37°C for 1 hour. After incubation, cells were spun down, supernatants were aspirated, and resuspended in 250 ul 10% SDS then vortexed and incubated for 30 minutes at 65°C. After incubating the samples on ice for 5 minutes, 100 ul of 5M potassium acetate, pH7.5 was added and samples incubated on ice for 1h. Tubes were spun at 14000 RPM for 15 minutes and the supernatant was transferred to a new tube. Then 400 ul of ice cold 95% EtOH was added, vortexed briefly, and spun for 15 minutes at 14000 RPM. The supernatants were decanted and DNA pellet dried at 30°C for 15 minutes, then it was dissolved in 100 ul of ultra-pure water, and placed in a mixer for 20 minutes at 2000 RPM. The extracted genomic DNA was used to perform PCR with high-fidelity polymerase (Takara Bio Inc) to confirm the transgene expression by Sanger sequencing.

### Yeast RNA extraction

RNA was extracted from yeast cells as previously described (107). Briefly, 1 mL of yeast culture was pelleted and then resuspended in 400 ul of AE buffer (50 mM, pH 5.2 of NaOAc, 10Mm EDTA). After the addition of 40ul of 10%SDS and 400ul of PCA, samples were incubated at 65°C for 10 minutes. After a 5 min incubation on ice, samples were placed in phase lock gel tubes, centrifuged at 14,000 RPM for 5 minutes and centrifuged again using the same conditions following another chloroform addition; this chloroform wash was repeated again, and the samples were centrifuged one final time. The aqueous phase was transferred to a new 1.5 ml tube, 50ul of 3M sodium acetate ph 5.2 was added, followed by 2 volumes of 100% ethanol. Samples were centrifuged at 14,000 RPM for 15 minutes, the ethanol was removed and the pellet washed with 70% ethanol, then centrifuged at 14,000 RPM for 5 minutes, ethanol was removed, and samples were air dried. The pellet was resuspend in RNAse-free water, and then vortexed. To remove any potential DNA contamination, 1ug of RNA was treated with DNAse turbo following manufacturer instructions. RNA was extracted after DNAse treatment with phenol/chloroform/isoamyl alcohol and then ethanol precipitation. RNA concentration was measured using the NanoDrop (ThermoFisher).

### Yeast spot assay

One milliliter of freshly harvested cells grown overnight in 10mL of YEPD were centrifuged, washed in ultra-pure water and sonicated. The cells were then diluted to a concentration of 1x107. Serial ten fold (up to 5 times) were prepared on both Galactose (Gal) and non-galactose plates. The plates were incubayed at 30° C for 3 days.

### Yeast growth curve

Yeast was grown in YEPD overnight. When the OD660 reached approximately 0.5, transgene activation was induced by adding galactose dissolved in water to a final concentration of 2% or water as control. For each time point samples were collected for cell count and RNA extraction.

### RNA sequencing and analysis

RNA-seq data from the ENCODE project for shQKI and shNT or shSF1 and shNT (56, 108) were downloaded from GEO and analyzed as previously described (89) using Vast-tools (57, 58) or rMATS (61) to compare alternative splicing observed in shQKI relative to shNT, or shSF1 relative to shNT

The subsequent Vast-tools datafiles were filtered by enlisting a cutoff of dPSI > |10| and MVdPSI95 > 0 to measure alternatively spliced events that changed significantly upon either QKI or SF1 knockdown compared to control. For “co-regulated” exons, the above cutoff was required for either shQKI compared to control or shSF1 compared to control, but in both datasets the MVdPSI95 had to be > 0. rMATS analysis and rMAPS2 (60, 109) motif enrichments were done using default conditions and with custom motifs added to measure various bp-like and potential SF1 binding motifs (ACT[ACTG]AG, [ACTG]CT[AG][CT], TAA[CT], TAA[CT]T[ACTG]A[CT], TACTAAC, TACTAA, ACTAA[CT], TACTAA[CT], CTAAC[ACG]).

Strand-specific RNAseq libraries were prepared from 1ug of yeast total RNA using NEBNext poly(A) mRNA Magnetic Isolation module (NEB, E7490) and NEBNext Ultra II Directional RNA Library Prep kit for Illumina (NEB, E7760) following the manufacturer’s recommended procedure. The six libraries were pooled in equal molar concentration and sequenced on Illumina NextSeq 550 for PE 150 base pair sequencing yielding about 30M paired end reads each. The associated datafiles have been uploaded to GEO under accession GSE273838 and are accessible using reviewer token urerikkgljkddcp The RNAseq reads were filtered for low quality bases and trimmed of adapter sequences using trimmomatics (v.0.39). The trimmed reads were aligned to the yeast reference genome SacCer3 and a custom yeast annotation file which accounts for all yeast introns (46) (and allows measurement of unspliced, spliced and intronless transcripts) using kallisto (v.0.50) to generate the abundance.tsv file for either normalized (TPM) or total (counts) reads for each sample. The TPM file was used as input for Deseq2 analysis (v1.42.1) to identify changes in transcript abundance (abundance cutoff TPM > 0.2 and significance cutoff *P* > 0.1; Supplemental Table S6), and the read counts file was used to calculate the percentage of unspliced RNA (percent unspliced = (unspliced/(unspliced+spliced))*100) for each transcript that was expressed (base mean > 100) and statistical significance was measured using the Student’s t-test (double check w/Jose) with a change in percent unspliced *P* < 0.1 considered significant (Supplemental Table S7).

Motif analysis of both the yeast and HepG2 RNA-seq dataset were performed using SEA (59) and the former used 80 nt of intron sequence upstream of the 3’ss of yeast introns, 38 of which increased (*P* < 0.1) upon ectopic Qki5 expression compared to the parental control (this represents 38 of the 41 we observed that increased in inclusion upon Qki5 expression, as the other two were located on ChrM and did not overlap with “Talkish Standard Introns” (74) which was a requirement for out motif analysis), compared to a background set of 106 introns that were detectable (base mean > 100) but unchanged (P > 0.2, change in percent unspliced < |1|) upon ectopic Qki5 expression compared to the parental control. For the HepG2 intron set, we extracted the intron sequence that began 20 nt upstream of the 3’ss (to exclude analyzing potential differences in 3’ss or pY tracts) and spanned 60 nt upstream of the 3’ss of introns that were co-regulated by QKI and SF1 (dPSI > |10| and MVdPSI at 95% confidence interval > 0 in either dataset or the other and at least MVdPSI at 95% confidence interval > 0 in both datasets, as determined by Vast-tools) and performed SEA, and then also performed an identical analysis but using 1000 introns that were detectable (base mean > 100) but unchanged in either shQKI relative to shNT or shSF1 relative to shNT (dPSI < |1|, MVdPSI = 0). In both cases, the motif set that was interrogated was from Ray et al 2013 (110).

### RNA affinity chromatography

Tobramycin RNA affinity chromatography was performed as previously described (63) with several minor modifications. Briefly, the aptamer and RAI14 WT and mutant RNAs were produced after the vectors were digested with Not1, with HiScribe T7 High Yield RNA Kit (NEB). Then 120 picomoles of RNA of the aptamer and RAI14 mutants, and 200pMoles of wt RAI14 were heated in RNA binding buffer (20 mM Tris-HCl, 1 mM CaCl2:, 1 mM MgCl2, 300 mM KCl, 0.1 mg/ml tRNA, 0.5 mg/ml BSA, 0.01% NP-40, 0.2 mM DTT) at 95 °C for 5 minutes and transferred to RT for 30 minutes. Samples were incubated with 60µl of tobramycin-coupled sepharose matrix at 4°C for 2 hours with head-over-tail rotation. The beads where washed three times with washing buffer (20 mM Tris-HCl, 1 mM CaCl2, 1 mM MgCl2, 145 mM KCl, 0.1% NP-40, 0.2 mM DTT) and incubated with a 285 ul solution of 32% of C2C12 nuclear extracts (as previously described (111)), 32 mM KCL, 2 mM MgCl2, 2 mM ATP and 20 mM creatine phosphate (the reactions were also performed without MgCl2, ATP and CP in the nuclear extract solution) for 7.5, 15 and 30 min at 30C with head over tail rotation. After nuclear extract incubation, the matrix was washed three times with a higher salt concentration of washing buffer (150 mM KCl). The beads were eluted with 5 mM of tobramycin in 20 mM Tris-HCl, 1 mM CaCl2, 1 mM MgCl2, 145 mM KCl, 2 mM MgCl2, 0.2 mM DTT in a 125 ul solution. The protein eluate was precipitated with acetone and suspended in LDS sample buffer for WB and in 5% SDS, 50 mM TEAB pH 7.1 for MS analysis.

### Protein precipitation

Acetone was used to precipitate the proteins from the RNA affinity chromatography-eluted samples. Four times the sample volume of cold acetone was added to each sample and vortexed. Samples were incubated for 60 minutes at −20°C for one hour, following a 10 minutes centrifugation at 14,000 RPM at 4C. The acetone was decanted and samples were air dried for 15 minutes. Pellets were resuspended with 1x laemmli buffer for western blot or with 5% SDS, 50 mM TEAB pH 7.1 for liquid chromatography with tandem mass spectrometry.

### Protein digestion

The samples were prepared as previously described (112). Briefly, 25ug of protein from the above were reduced with 10 mM Tris(2-carboxyethyl) phosphine (TCEP) (77720, Thermo) and incubated at 65 °C for 10 min. The sample was then cooled to room temperature and 1 μl of 500 mM iodoacetamide acid was added and allowed to react for 30 min in the dark. Then, 3.3 μl of 12% phosphoric acid was added to the protein solution followed by 200 ul of binding buffer (90% Methanol, 100 mM TEAB pH 8.5). The resulting solution was added to S-Trap spin column (protifi.com) and passed through the column using a bench top centrifuge (60s spin at 1,000 x *g*). The spin column is washed with 150 ul of binding buffer and centrifuged. This is repeated two times. 30 ul of 20 ng/ul trypsin is added to the protein mixture in 50 mM TEAB pH 8.5, and incubated at 37°C overnight. Peptides were eluted twice with 75ul of 50% acetonitrile, 0.1% formic acid. Aliquots of 20 ul of eluted peptides were quantified using the Quantitative Fluorometric Peptide Assay (Pierce, Thermo Fisher Scientific). Eluted volume of peptides corresponding to 5.5 ug of peptides are dried in a speed vac and resuspended in 27.5 ul 1.67% acetonitrile, 0.08% formic acid, 0.83% acetic acid, 97.42% water and placed in an autosampler vial.

### Data dependent acquisition NanoLC MS/MS Analysis.

Peptide mixtures were analyzed by nanoflow liquid chromatography-tandem mass spectrometry (nanoLC-MS/MS) using a nano-LC chromatography system (UltiMate 3000 RSLCnano, Dionex), coupled on-line to a Thermo Orbitrap Fusion mass spectrometer (Thermo Fisher Scientific, San Jose, CA) through a nanospray ion source (Thermo Scientific) similar to as we have described previously (113). A trap and elute method was used. The trap column was a C18 PepMap100 (300um X 5mm, 5um particle size) from ThermoScientific. The analytical column was an Acclaim PepMap 100 (75um X 25 cm) from (Thermo Scientific). After equilibrating the column in 98% solvent A (0.1% formic acid in water) and 2% solvent B (0.1% formic acid in acetonitrile (ACN)), the samples (2 µl in solvent A) were injected onto the trap column and subsequently eluted (400 nL/min) by gradient elution onto the C18 column as follows: isocratic at 2% B, 0–5 min; 2% to 24% B, 5–86 min; 24% to 44% B, 86–93 min; 44% to 90% B, 93–95 min; 90% B for 1 minute, 90% to 10% B, 96–98 min; 10% B for 1 minute 10% to 90% B, 99–102 min 90% to 4% B; 90% B for 3 minutes; 90% to 2%, 105–107 min; and isocratic at 2% B, till 120 min.

All LC-MS/MS data were acquired using XCalibur, version 2.5 (Thermo Fisher Scientific) in positive ion mode using a top speed data-dependent acquisition (DDA) method with a 3 sec cycle time. The survey scans (*m/z* 350–1500) were acquired in the Orbitrap at 120,000 resolution (at *m/z* = 400) in profile mode, with a maximum injection time of 100 msec and an AGC target of 400,000 ions. The S-lens RF level is set to 60. Isolation is performed in the quadrupole with a 1.6 Da isolation window, and CID MS/MS acquisition is performed in profile mode using rapid scan rate with detection in the ion-trap, with the following settings: parent threshold = 5,000; collision energy = 32%; maximum injection time 56 msec; AGC target 500,000 ions. Monoisotopic precursor selection (MIPS) and charge state filtering were on, with charge states 2–6 included. Dynamic exclusion is used to remove selected precursor ions, with a +/− 10 ppm mass tolerance, for 15 sec after acquisition of one MS/MS spectrum.

### DDA Database Searching

Tandem mass spectra were extracted and charge state deconvoluted by Proteome Discoverer (Thermo Fisher, version 2.2.0388). Charge state deconvolution and deisotoping were not performed. All MS/MS samples were analyzed using Sequest (Thermo Fisher Scientific, San Jose, CA, USA; in Proteome Discoverer 2.5.0.402). Sequest was set up to search the conanical mouse proteome and a contaminant database cRAP assuming the digestion enzyme trypsin. Sequest was searched with a fragment ion mass tolerance of 0.60 Da and a parent ion tolerance of 10.0 PPM. Carbamidomethyl of cysteine was specified in Sequest as a fixed modification. Deamidated of asparagine and glutamine, oxidation of methionine and acetyl of the n-terminus were specified in Sequest as variable modifications.

### Criteria for Protein Identification

Scaffold (version Scaffold_4.11.1, Proteome Software Inc., Portland, OR) was used to validate MS/MS based peptide and protein identifications. Peptide identifications were accepted if they could be established at greater than 95.0% probability by the Scaffold Local FDR algorithm. Protein identifications were accepted if they could be established at greater than 99.0% probability and contained at least 2 identified peptides. Protein probabilities were assigned by the Protein Prophet algorithm (114). Proteins that contained similar peptides and could not be differentiated based on MS/MS analysis alone were grouped to satisfy the principles of parsimony. Proteins sharing significant peptide evidence were grouped into clusters. The resulting normalized spectral counts from the input NE, each RAC substrate (WT, upDEL, dnDEL, 2xDEL, and APT Only control) were obtained, and those values were used to calculated background-corrected levels of enrichment relative to nuclear extract ((NSC_RAC_ – NSC_APT_)/NSC_NE_). We used those calculations to construct the heatmap shown in Fig 4B and generate the counts shown in Fig 4C, by including proteins with experimental LC-MS/MS evidence of being members of early spliceosomes (E-complex or 17S U2 snRNP (64–66)), and that had a positive value of background-corrected levels of enrichment relative to nuclear extract in at least one of the 4 RAC substrates tested. See Supplemental Table S4.

### Data independent acquisition NanoLC MS/MS Analysis

Peptide mixtures were analyzed by nanoflow liquid chromatography-tandem mass spectrometry (nanoLC-MS/MS) using a nano-LC chromatography system (UltiMate 3000 RSLCnano, Dionex), coupled on-line to a Thermo Orbitrap Eclipse mass spectrometer (Thermo Fisher Scientific, San Jose, CA) through a nanospray ion source. A direct injection method is used onto an analytical column; Aurora (75um X 25 cm, 1.6 µm) from (ionopticks). After equilibrating the column in 98% solvent A (0.1% formic acid in water) and 2% solvent B (0.1% formic acid in acetonitrile (ACN)), the samples (2 µL in solvent A) were injected (300 nL/min) by gradient elution onto the C18 column as follows: isocratic at 2% B, 0–10 min; 2% to 27% 10–98 min, 27% to 45% B, 98–102 min; 45% to 90% B, 102–103 min; isocratic at 90% B, 103–104 min; 90% to 15%, 104–106 min; 15% to 90% 106–108 min; isocratic for two minutes; 90%-2%, 110–112 min; and isocratic at 2% B, till 120 min.

All LC-MS/MS data were acquired using an Orbitrap Eclipse in positive ion mode using a data-independent acquisition (DIA) method with a 16Da windows from 400–1000 and a loop time of three seconds. The survey scans (m/z 350–1500) were acquired in the Orbitrap at 60,000 resolution (at m/z = 400) in centroid mode, with a maximum injection time of 118 msec and an AGC target of 100,000 ions. The S-lens RF level was set to 60. Isolation was performed in the quadrupole, and HCD MS/MS acquisition was performed in profile mode using the orbitrap at a resolution of 30000 using the following settings: collision energy = 33%, IT 54ms, AGC target = 50,000. These conditions were duplicated to create six gas-phase fractions of the NE sample using 4Da fully staggered windows in 100 m/z increments from 400–1000 m/z, as described (115).

### DIA Database Searching

The raw data was demultiplexed to mzML with 10 ppm accuracy after peak picking in MSConvert (116). The resulting mzML files were searched in MSFragger (117) and quantified via DIA-NN (https://github.com/vdemichev/DiaNN) using the following settings: peptide length range 7–50, protease set to Trypsin, 2 missed cleavages, 3 variable modifications, clip N-term M on, fixed C carbamidomethylation, variable modifications of methionine oxidation and n-terminal acetylation, MS1 and MS2 accuracy set to 20 ppm, 1% FDR, and DIANN quantification strategy set to Robust LC (high accuracy). The files were searched against a database of human acquired from Uniprot (18^th^ December, 2023). The gas-phase fractions were used only to generate the spectral library, which was used for analysis of the individual samples.

Statistical analysis was performed using Fragpipe-Analyst (118) using an R script based on the ProteinGroup.txt file produced by DIA-NN. First, contaminant proteins, reverse sequences and proteins identified “only by site” were filtered out. In addition, proteins that have been only identified by a single peptide and proteins not identified/quantified consistently in same condition are removed as well. The DIA data was converted to log_2_ scale, samples were grouped by conditions, and missing values were not imputed. Protein-wise linear models combined with empirical Bayes statistics were used for the differential expression analyses. The limma package from R Bioconductor was used to generate a list of differentially expressed proteins for each pair-wise comparison. A cutoff of the adjusted *P*-value of 0.05 (Benjamini-Hochberg method) along with an absolute log2 fold change of 1 has been applied to determine significantly regulated proteins in each pairwise comparison. We also used the E/U2 list to focus our analysis on early spliceosome proteins, and expanded this to include a RBP list (M Weirauch and Q Morris via personal communication and manuscript under review) to assess non-spliceosome-annotated RBP interactions. The E/U2 or RBPs shown in Fig 4E were included if 1) spectral counts were detected in both the numerator and denominator RAC substrates, 2) log2 fold change ≥ |0.2| and *P*-value was < 0.01 in at least one of the three comparison groups, and 3) data were present for each comparison (no zero or undetectable values). See Supplemental Table S5.

## Supplementary Material

Supplement 1

Supplement 2

Supplement 3

Supplement 4

Supplement 5

Supplement 6

Supplement 7

Supplement 8 **Supplemental Figure S1:** Table indicating the numbers of alternative splicing events that passed cutoff for significance (dPSI ≥ |10| and MVdPSI > 0) in Fig 1B.
**Supplemental Figure S2:** Western blot and RT-PCR of proteins and RNA extracted from WT HEK293 cells transfected with tdTomato, WT myc:Qki5, MT myc:Qki5 and SF1. The top panel shows a western blot probed with anti-SF1 (magenta), anti-PanQKI (green, middle) and anti-Gapdh (magenta, bottom). Below RT-PCR products analyzed on a Bioanalyzer from RNA extracted from transfected WT HEK 293 cells with mean percent included and ± standard deviation bellow (**P <0.01,***P < 0.001). The results shown are representative of 3 biological replicates.
**Supplemental Figure S3:** Agarose gel showing PCR amplification in the absence (−) or presence (+) of reverse transcriptase. PCR was performed with RNA from C2C12 cells transfected with RAI14 reporter plasmids.
**Supplemental Figure S5:** A. Growth curve of Gal- inducible GFP, mtQki5 and WT Qki5 BY4741 yeast cells grown in the absence (blue) or presence of galactose (orange). The y-axis shows the log_2_ number of cells and the horizontal axis the time point (in hours) that cells were collected and counted. B. Representative phas contrast microscopy showing images of GFP, mtQki5 and WTQki5 expressing yeast cells (1000x) at 24 hours after galactose induction. Inset shows enlarged regions to provide more detailed cell morphology information. C. RT-PCR of parental BY4741 or BY4741 with the Qki5 transgene 4h after galactose induction, measuring various intron-retention events predicted upon ectopic Qki5 expression (with exception of control HAC1) using primers that span intron-exon junction for each target and analyzed on an agarose gel (n = 3 per condition).
