# Supplementary material for: An ancient competition for the conserved branchpoint sequence influences physiological and evolutionary outcomes in splicing
Source: bioRxiv. 2024 Oct 9:2024.10.09.617384. Preprint. [Version 1] doi: 10.1101/2024.10.09.617384 (PMC11483029; doi:10.1101/2024.10.09.617384)

SUPPLEMENTAL FIGURE S1

shRNA	AltEX		RI	
	up	dn	up	dn
shQKI	477	243	1783	100
shSF1	351	694	358	727

SUPPLEMENTAL FIGURE S2

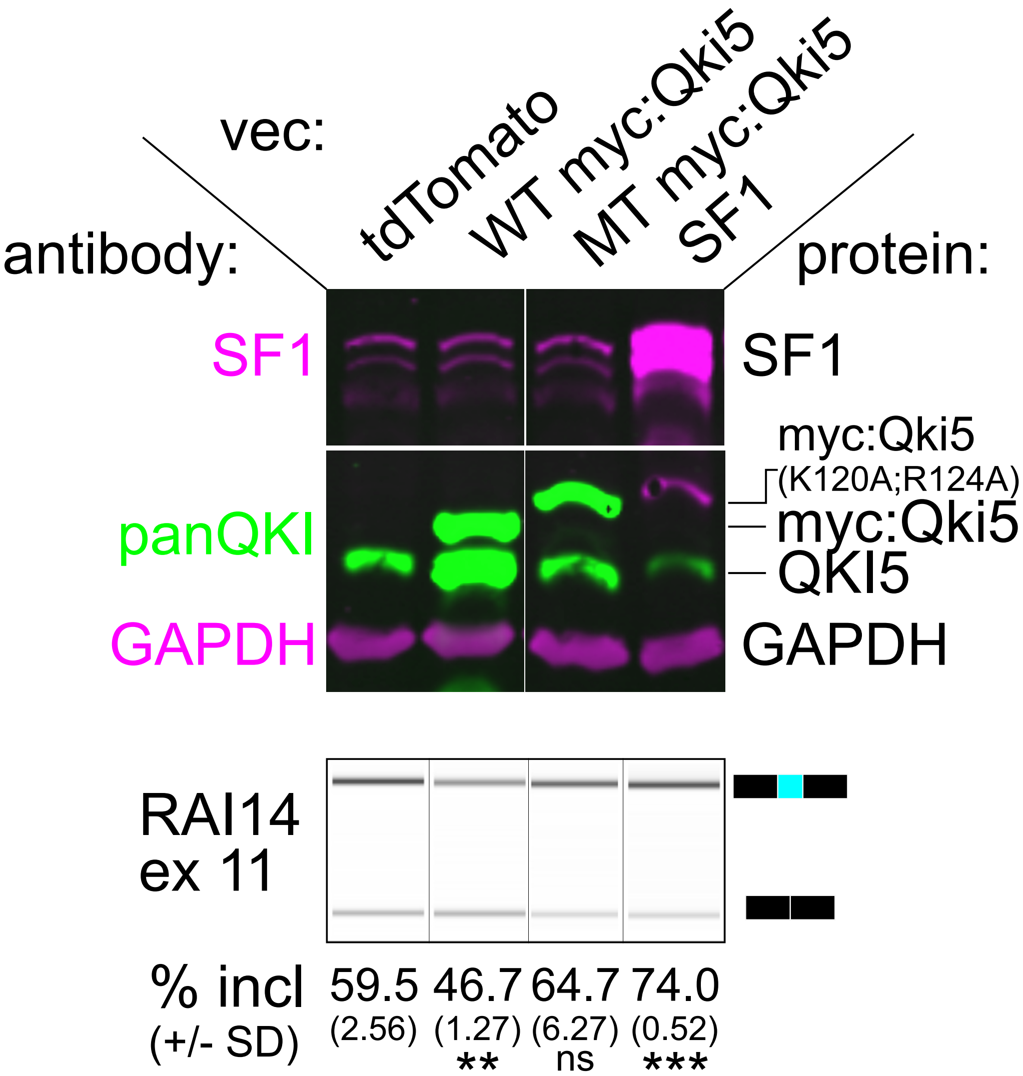

SUPPLEMENTAL FIGURE S3

DUP-RAI14 constructs +/-RT

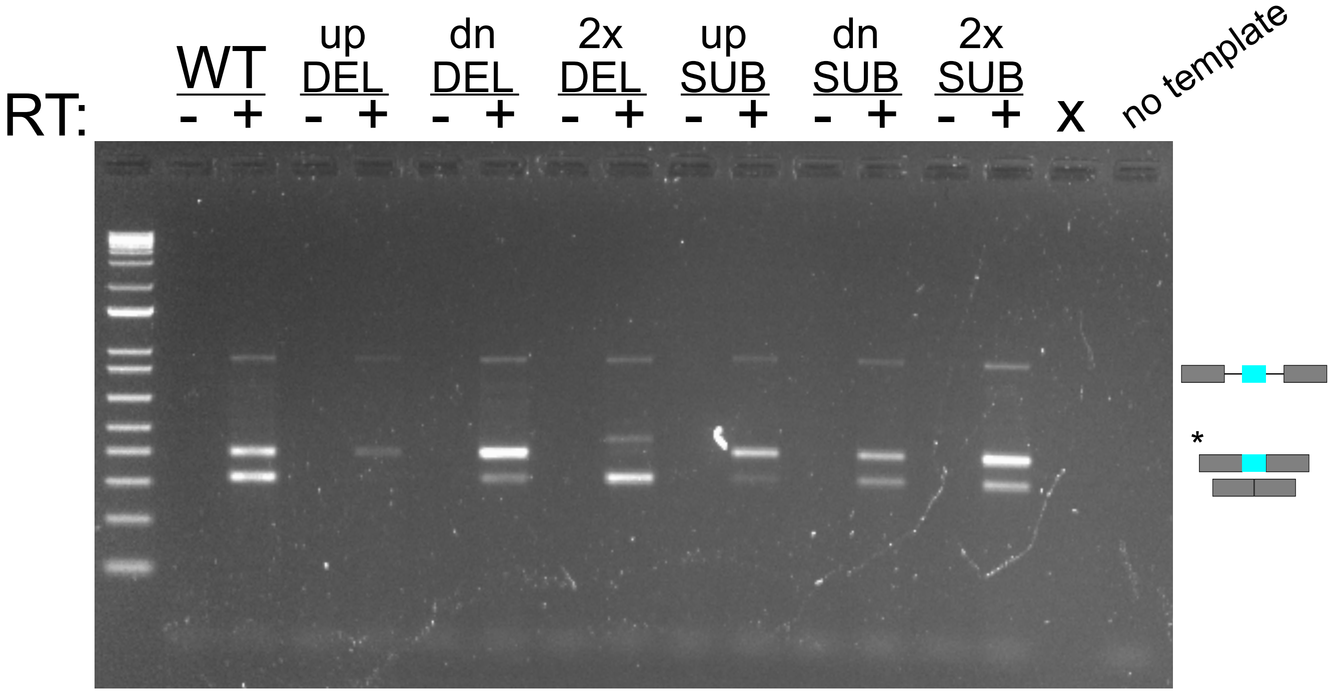

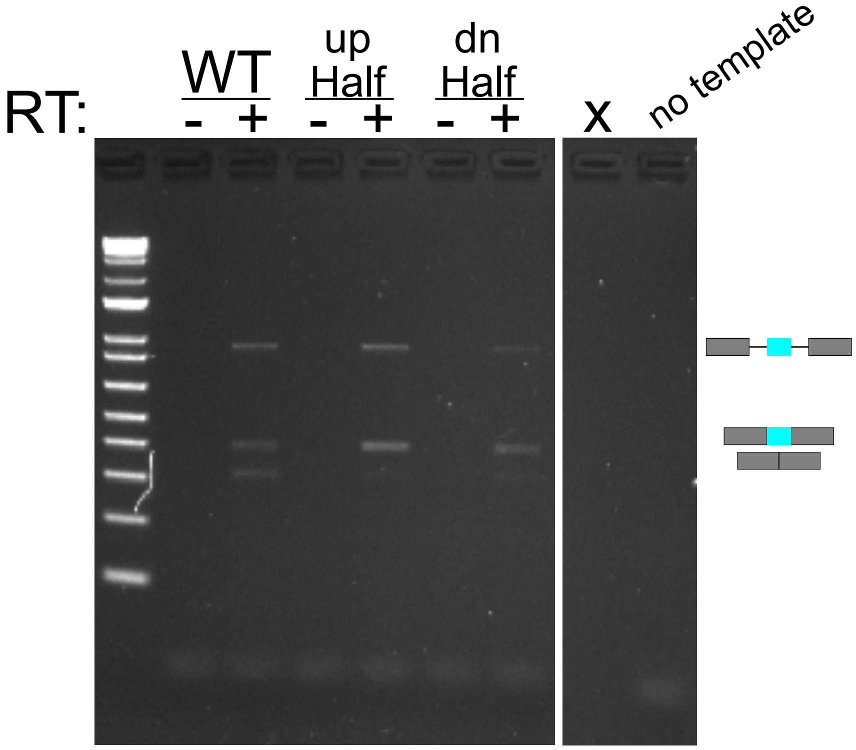

SUPPLEMENTAL FIGURE 5

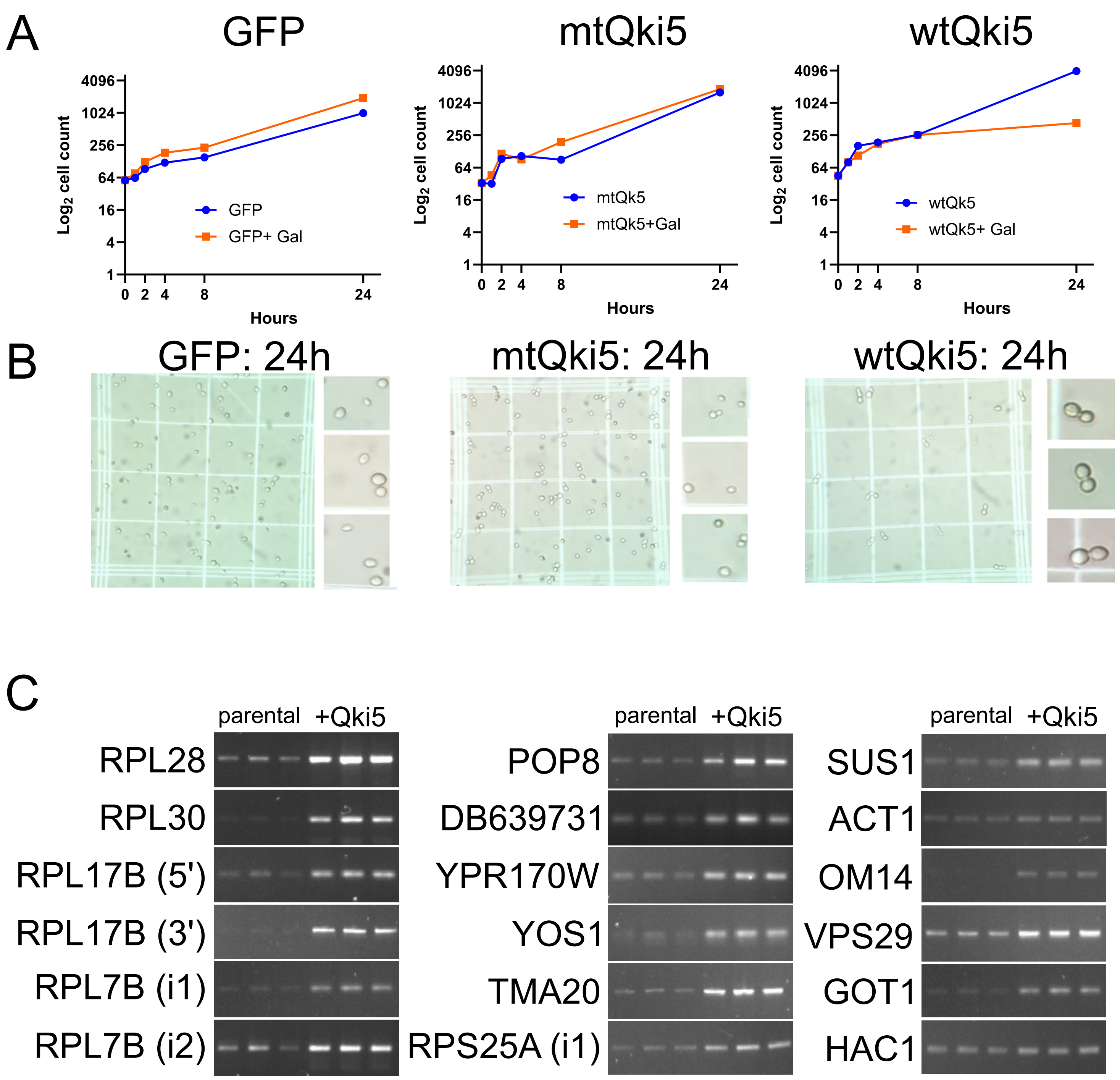

Supplement: Supplement 8 — Supplemental Figure S1: Table indicating the numbers of alternative splicing events that passed cutoff for significance (dPSI ≥ |10| and MVdPSI > 0) in Fig 1B. Supplemental Figure S2: Western blot and RT-PCR of proteins and RNA extracted from WT HEK293 cells transfected with tdTomato, WT myc:Qki5, MT myc:Qki5 and SF1. The top panel shows a western blot probed with anti-SF1 (magenta), anti-PanQKI (green, middle) and anti-Gapdh (magenta, bottom). Below RT-PCR products analyzed on a Bioanalyzer from RNA extracted from transfected WT HEK 293 cells with mean percent included and ± standard deviation bellow (**P <0.01,***P < 0.001). The results shown are representative of 3 biological replicates. Supplemental Figure S3: Agarose gel showing PCR amplification in the absence (−) or presence (+) of reverse transcriptase. PCR was performed with RNA from C2C12 cells transfected with RAI14 reporter plasmids. Supplemental Figure S5: A. Growth curve of Gal- inducible GFP, mtQki5 and WT Qki5 BY4741 yeast cells grown in the absence (blue) or presence of galactose (orange). The y-axis shows the log2 number of cells and the horizontal axis the time point (in hours) that cells were collected and counted. B. Representative phas contrast microscopy showing images of GFP, mtQki5 and WTQki5 expressing yeast cells (1000x) at 24 hours after galactose induction. Inset shows enlarged regions to provide more detailed cell morphology information. C. RT-PCR of parental BY4741 or BY4741 with the Qki5 transgene 4h after galactose induction, measuring various intron-retention events predicted upon ectopic Qki5 expression (with exception of control HAC1) using primers that span intron-exon junction for each target and analyzed on an agarose gel (n = 3 per condition). [file NIHPP2024.10.09.617384v1-supplement-8.pdf]
